# Characteristics of the gait adaptation process due to split-belt treadmill walking under a wide range of right-left speed ratios in humans

**DOI:** 10.1371/journal.pone.0194875

**Published:** 2018-04-25

**Authors:** Hikaru Yokoyama, Koji Sato, Tetsuya Ogawa, Shin-Ichiro Yamamoto, Kimitaka Nakazawa, Noritaka Kawashima

**Affiliations:** 1 Department of Life Sciences, Graduate School of Arts and Sciences, University of Tokyo, Meguro, Tokyo, Japan; 2 Department of Rehabilitation for the Movement Functions, Research Institute, National Rehabilitation Center for Persons with Disabilities, Tokorozawa, Japan; 3 Japan Society for the Promotion of Science, Chiyoda, Tokyo, Japan; 4 Department of Bioscience and Engineering, Graduate School of Engineering and Science, Shibaura Institute of Technology, Minuma, Saitama, Japan; West Virginia University, UNITED STATES

## Abstract

The adaptability of human bipedal locomotion has been studied using split-belt treadmill walking. Most of previous studies utilized experimental protocol under remarkably different split ratios (e.g. 1:2, 1:3, or 1:4). While, there is limited research with regard to adaptive process under the small speed ratios. It is important to know the nature of adaptive process under ratio smaller than 1:2, because systematic evaluation of the gait adaptation under small to moderate split ratios would enable us to examine relative contribution of two forms of adaptation (reactive feedback and predictive feedforward control) on gait adaptation. We therefore examined a gait behavior due to on split-belt treadmill adaptation under five belt speed difference conditions (from 1:1.2 to 1:2). Gait parameters related to reactive control (stance time) showed quick adjustments immediately after imposing the split-belt walking in all five speed ratios. Meanwhile, parameters related to predictive control (step length and anterior force) showed a clear pattern of adaptation and subsequent aftereffects except for the 1:1.2 adaptation. Additionally, the 1:1.2 ratio was distinguished from other ratios by cluster analysis based on the relationship between the size of adaptation and the aftereffect. Our findings indicate that the reactive feedback control was involved in all the speed ratios tested and that the extent of reaction was proportionally dependent on the speed ratio of the split-belt. On the contrary, predictive feedforward control was necessary when the ratio of the split-belt was greater. These results enable us to consider how a given split-belt training condition would affect the relative contribution of the two strategies on gait adaptation, which must be considered when developing rehabilitation interventions for stroke patients.

## Introduction

Locomotor adaptation is a remarkable ability of humans with important implications for meeting the demands of the environment. For example, when we encounter changes on road surfaces (e.g., slippery) and body mechanics (e.g., injury), “default” locomotion movement is perturbed and then modified in accordance with the internal/external environment. To reveal the adaptability of human bipedal locomotion, split-belt treadmill walking has been extensively utilized as a useful experimental model over the past decades [1–18]. In this model, subjects walk on two belts (left and right sides) independently moving on different speeds and acquire a new walking pattern to achieve energetically efficient walking behavior under the novel environment [13].

In the mid-2000s, previous studies have revealed that locomotor adaptation has two processes. Some parameters, such as stance time and stride length, exhibit quick adjustment at the initial adaptation period (several seconds), whereas other parameters, such as step length and double support time, exhibit relatively long-lasting adaptive process (several minutes) and subsequent aftereffects [1]. The two above-mentioned distinct adaptation processes can be regarded as reactive adjustment (short term, feedback) and predictive adaptations (longer term, feedforward), respectively,[1,19]. Existence of the two distinct types of processes in locomotor adaptation has been supported by several evidences that the reactive adjustment and predictive adaptation are contributed by different neural structures (i.e., the spinal cord and cerebellum, respectively) [9,20–22] (for details, see the following paragraphs). Additionally, Fujiki et al. [23] demonstrated that the two distinct adaptation processes were able to be reproduced by a walking control model composed of spinal model, which generate basic movement patterns and modulate them immediately in response to sensory inputs, and cerebellar model, which predictively modify movements based on movement error.

Regarding the control mechanisms of the two adaptation processes, it is assumed that different neural mechanisms are involved in the processes of locomotor adaptation. The reactive process would be automatically induced reflex system at the lower level of the central nervous system (CNS), i.e., the spinal cord and the brain stem. For example, studies on spinalized cats demonstrated that position and load inputs from the ankle and the hip joint quickly modify the timing of the stance-to-swing transition [24,25]. Regarding split-belt walking, a classic study demonstrated that spinalized cats can quickly adjust the swing and stance time on each leg to the speed difference between left and right belts [26]. Additionally, human infants present reactive adjustments during split-belt walking [20]. As the infants’ developmental stage was considered to be before the myelination of their corticospinal pathways [27], it was suggested that reactive adjustments are controlled primarily at the spinal level. Subjects with cerebellar damage can also modify their locomotion patterns through the reactive feedback process [9].

Upon the emergence of the predictive feedforward control (i.e, slow adaptive change and subsequent aftereffects), the cerebellum has critical roles for the adaptation processes in humans [9] and cats [22]. Morton and Bastian [9] demonstrated that predictive feedforward control was impaired in patients with cerebellar damage. Similarly, cats whose cerebellar function was artificially impaired had disrupted predictive control [22].

The cerebellum plays an important role in sensory processing and modifying ongoing movement patterns [28,29]. Regarding sensory perception during split-belt walking, a recent study by Hoogkamer et al. [12] showed that the perception threshold of speed difference of the split-belt is around the 1:1.2 ratio. Thus far, previous split-belt walking adaptation studies used larger speed ratios than the perception threshold (i.e., larger than 1:2) [1,4,7,8]. Based on the knowledge that the predictive process of locomotor adaptation is related to sensory processing, the contribution of the predictive process to locomotor adaptation may be insignificant in smaller speed ratio of split-belt walking. Meanwhile, assuming that the reactive process is critically involved in the reflective mechanisms of the spinal cord and the brain stem, the size of reactive adjustment may have a linear relationship with the speed ratio of the split-belt. However, there are limited perspectives on the systematic gradation of split-belt adaptation from small to large speed ratios. Systemization of split-belt adaptation in a small speed ratio can provide information on the critical point of emergence of long-lasting learning memory. Thus, the information leads to the construction of locomotor training regimens in less perturbed conditions for patients.

The purpose of this study was therefore to observe behavioral nature of the adaptive process in accordance with the split-belt speed ratio between the left and right sides, based on the systematic evaluation of locomotor adaptation among five different speed ratios of the split-belt treadmill. We focused on the relative contribution of the two forms of adaptation processes (reactive and predictive) to split-belt walking adaptation. We hypothesized that (1) the degree of reactive adjustment has a linear relationship to the ratio of split-belt and that (2) the contribution of the predictive process to locomotor adaptation is relatively minor in smaller speed ratio of split-belt walking.

## Materials and methods

### Participants

Ten healthy young male subjects (28.1±4.94 yrs) were recruited from a group of volunteers in Research Institute of National Rehabilitation Center for Persons with Disabilities (Japan). No participants had any history of neurological or musculoskeletal disorders. All subjects gave written informed consent for their participation in this study, which were approved by the local ethics committee of the National Rehabilitation Center for Persons with Disabilities, Japan. All of the experimental procedures were in accordance with the Declaration of Helsinki.

### Experimental procedures

**Fig 1:**
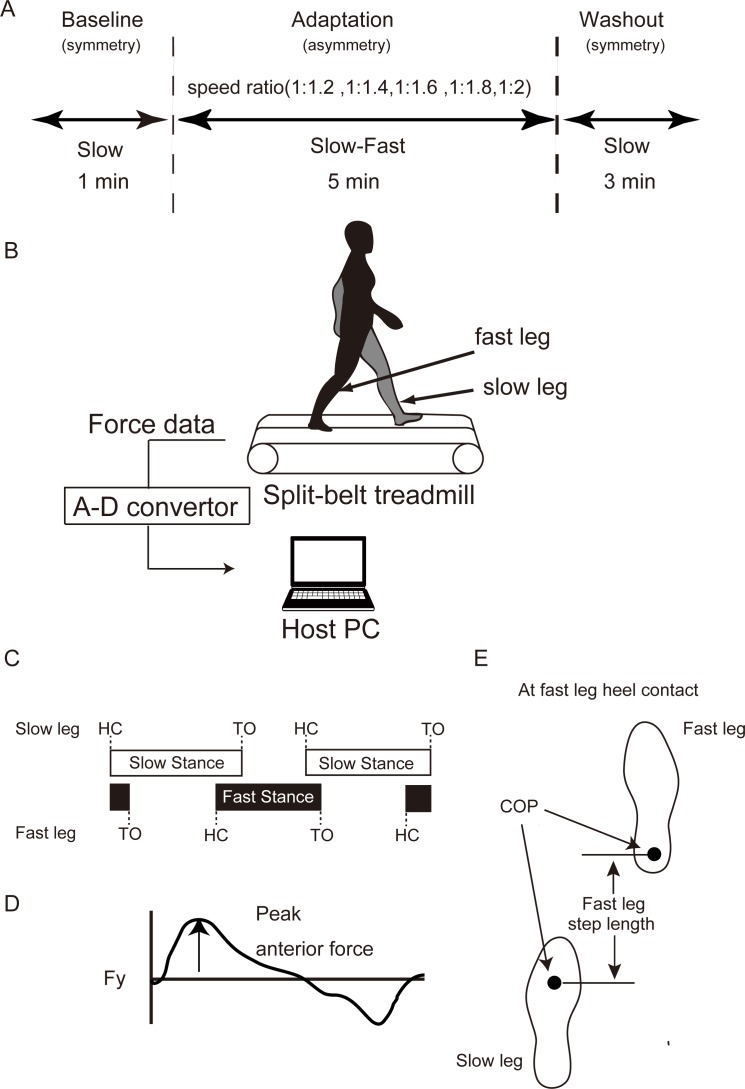
Experimental paradigm, setup and calculated gait parameters. (A): Experimental paradigm of split-belt treadmill adaptation under five different split-belt ratio conditions. (B): Experimental setup. Three orthogonal ground reaction forces (GRF) were recorded bilaterally. (C-E): Gait parameters calculated in the present study. (C): Schematic representation of the gait cycle and the timing of heel contact (HC), toe off (TO), and stance time. Horizontal bars represent the stance time (HC to TO) of the slow (white bars) and fast legs (black bars). (D): Waveform of the anteroposterior GRF. The peak value of the anterior braking force in each gait cycle was calculated. (E): Foot placements and their center of pressures (COP) at the timing of HC on the fast leg. Step length was defined as the length between the right and left COPs at the time of HC.

We applied an experimental paradigm established by Bastian and colleagues [1,4]. The protocol is shown in Fig 1A. In the present study, we performed five different speed ratio conditions of the split-belt adaptation trials. Subjects walked on a split-belt treadmill (Bertec, Columbus, OH, USA). Depending on the time period, the treadmill was operated in either of the following conditions: “tied-belt condition” (two belts moving at the same speed) or “split-belt condition” (moving at different speeds) modes. The fast- and slow-moving sides in the adaptation period were defined as the “fast side” and “slow side,” respectively. The fast and slow sides for the split-belt walking were the right and left legs, respectively, in common across all participants and conditions. First, the treadmill velocity was set at 1.0 m/s during the baseline period (1 min). Then, two belt speeds were set to one of the five speed ratios (1:1.2, 1:1.4, 1:1.6, 1:1.8, and 1:2) during the adaptation period (5 min). The slow belt was constantly set at 1.0 m/s, whereas the fast belt was set at 1.2, 1.4, 1.6, 1.8, or 2.0 m/s. Subsequently, the belt condition was again set at 1.0 m/s during the washout period (3 min). All subjects repeatedly participated in all of the five speed ratio conditions of split-belt walking adaptation trials on consecutive five days (i.e. –24 hrs between conditions). The order of the conditions was randomized across the subjects.

### Data measurements and analysis

During the walking task, three dimensional ground reaction force (GRF) data (Fx, Fy, Fz) and moments (Mx, My, Mz) were recorded separately on the right and left sides from the force plates under each belt of the treadmill at 1000 Hz (Fig 1B). The GRF and moment data were analyzed on a stride-by-stride basis for both the fast and slow legs, respectively. From the low-pass filtered (zero-lag Butterworth filter, 4-Hz cut-off, fourth-order) vertical component of GRF (Fz), the timing of heel contact and toe off was determined (threshold: 20 N). Based on heel contact and toe off, stance time was calculated as the time from the heel contact of one leg to the subsequent toe off of the same leg (Fig 1C). Subsequently, the peak amplitude of the anterior braking force was calculated from the Fy components (Fig 1D). Our previous study [8] demonstrated that this GRF component exhibits a clear pattern of adaptation and subsequent aftereffects in split-belt walking adaptation in addition to several other gait parameters (e.g., step length, double support time) revealed by Bastian and colleagues [1]. Additionally, we calculated the step length from the anterior-posterior component of the center of pressure (COP) between the left and the right leg (Fig 1E) [30]. In the present study, step length was defined as the length between the COPs of the two legs at the timing of heel contact. Fast step length is calculated as the length between the two COPs at heel contact of the fast leg (Fig 1E) and vice versa for the slow leg. COP was calculated from the recorded GRFs and momemts [31].

From the three gait parameters (stance time, peak anterior force, and step length), we removed the data on the first two stride cycles in each period to minimize the influences of perturbation of treadmill acceleration/deceleration. Then, we calculated the fast leg, slow leg, and symmetry index values in these parameters (symmetry index = (fast leg–slow leg)/(fast leg + slow leg)). To compare among subjects, the fast and slow leg values were normalized to the average of the last 30 seconds of the baseline period. All data were then divided into 5-second bin means because the stride cycles are different depending on the subject.

Then, a linear regression was used to evaluate the relationships between the speed ratio of the two belts and reactive responses (i.e., the symmetry indices of at initial adaptation and initial washout).

Additionally, to evaluate the relationships between the amount of adaptation (i.e., last value–initial value at the adaptation period) and the subsequent aftereffects, a linear regression was carried out in each speed ratio of the split-belt. Then, to evaluate the distribution characteristics of individual data in the relationship, the 95% confidence ellipses from the individual data sets were calculated. Then, we calculated the seven characteristic parameters of the regression lines and ellipses: slope and correlation coefficients of the regression lines and major axis length, ellipticity (ratio of the major and minor axis lengths), central coordinates, and area of the ellipses. The parameters were transformed into Z scores for normalization. Then, to compare the characteristics regarding the relationship between the amount of adaptation and aftereffect, hierarchical cluster analysis (Ward’s method, Euclidian distance) was performed for the five speed ratios in the 14-dimensional index parameter space (i.e., the seven normalized parameters of step length and anterior force).

### Statistics

We analyzed the symmetry indices of the three gait parameters (stance time, step length, and anterior force) across the different periods throughout the experiment (baseline, initial adaptation, last adaptation, initial washout, and last washout). The baseline values for each parameter were calculated as the average of the last 10 seconds of baseline walking. The initial adaptation and washout values were calculated as the average of the first 10 seconds in each of these respective periods. Likewise, the last adaptation and washout values were the average of the last 10 seconds during adaptation and washout, respectively. One-way repeated-measures analyses of variance were used to analyze changes in each parameter across these five periods. Subsequently, post hoc analyses were performed using Bonferroni’s test. Data are presented as mean and standard error (mean ± SE). Statistical significance was accepted at *p* < 0.05.

## Results

### Time series changes of gait parameters

**Fig 2:**
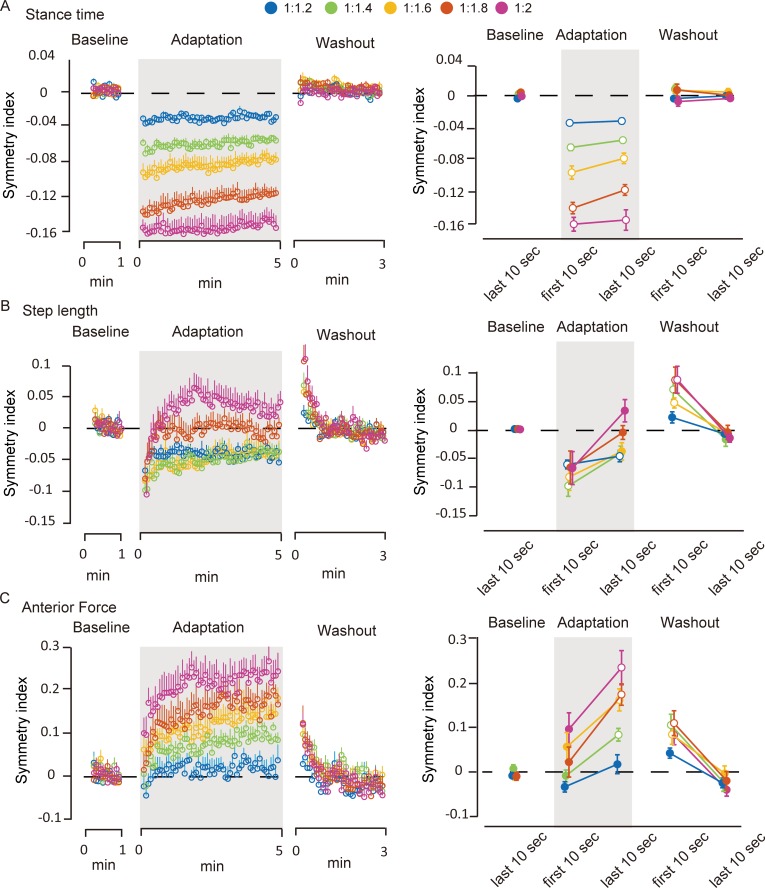
**Time series changes in the symmetry indices of gait parameters (A: stance time, B: step length, C: anterior force).** All data were normalized to those under the baseline condition. Left: Time series changes of the mean value of each 5-s bin. Right: Comparisons of each parameter at different time periods (baseline, first adaptation, last adaptation, first washout, and last washout). In the right panels, open circles denote significant differences against to the baseline period. Error bars indicate means ± SE. Significant differences were defined as *p* < 0.05.

Fig 2 shows the time series changes in the symmetry index of stance time (Fig 2A), step length (Fig 2B), and anterior force (Fig 2C) for the overall experimental protocol (left) and for the comparison among different time periods (right). In stance time (Fig 2A), at the initial adaptation period, the symmetry index showed significant decrease from that during the baseline in all conditions (*p* < 0.05 in all conditions, significant differences from the baseline are shown as open circles). During the 5-min adaptation period, the stance time symmetry gradually and only slightly returned toward the baseline. Thus, the symmetry index in all conditions remained small compared to the baseline level at the last adaptation period (*p* < 0.05 in all conditions). In the subsequent washout period, stance time quickly returned to symmetry in all conditions.

For the step length (Fig 2B), the symmetry index in all conditions decreased during the initial adaptation period compared with that during the baseline level (*p* < 0.05, in all conditions). The asymmetry steeply returned toward the baseline in the first minute and gradually became symmetric throughout the adaptation period in the four speed ratios except for the 1:1.2 condition, whereas the symmetry index in the 1:1.2 condition remained significantly different from that in the baseline period at the last adaptation period (*p* < 0.05). At the initial washout period, although the belt speed was tied, the step length exhibited significant asymmetry compared to that in the baseline period in all conditions except for the 1:1.2 condition (*p* < 0.05 in the 1:1.4, 1:1.6, 1:1.8, and 1:2 conditions). These aftereffects gradually decreased toward the baseline level within about the first 2 min.

For the anterior force (Fig 2C), there were no significant differences during the initial adaptation period compared to the baseline level. However, the symmetry index of the anterior force drastically became asymmetric in the first 1 min and moderately changed, lasting for the remaining 9 min during the adaptation period in the all speed ratios except for the 1:1.2 condition. As a result, the symmetry index was significantly different from those of the baseline in all speed ratios except for the 1:1.2 condition at the last adaptation period (*p* < 0.05 in 1:1.4, 1:1.6, 1:1.8 and 1:2). At the initial washout period, there were significant differences from those in the baseline period in all conditions except for the 1:1.2 condition (*p* < 0.05 in 1:1.4, 1:1.6, 1:1.8 and 1:2). As in the step length, the aftereffect gradually decreased toward symmetry within the first 2 min.

### Relationship between the speed ratio of split-belts and initial responses of locomotor parameters

**Fig 3:**
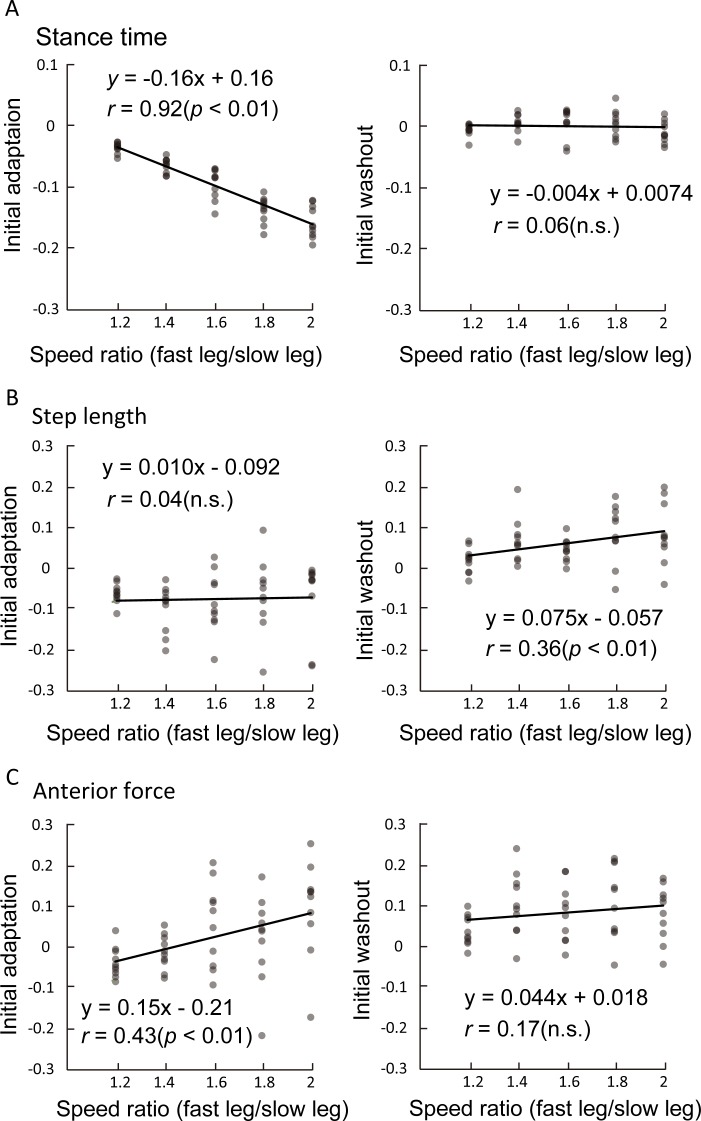
**Relevance of the speed ratio (x-axis) to the symmetry index of stance time (A), step length (B), and anterior force (C) at initial adaptation and initial washout (y-axis).** Each plot indicates individual data in each speed ratio. Regression lines and the correlation coefficients and their significance are presented.

Fig 3 shows the relationship between the speed ratio of the two belts and the symmetry index of stance time (Fig 3A), step length (Fig 3B), and anterior force (Fig 3C) at initial adaptation and initial washout. In stance time, although an extremely strong positive correlation was found at initial adaptation (*r* = 0.92, *p* < 0.001, Fig 3A, left), there is no correlation at initial washout (Fig 3A, right). In step length, contrary to stance time, no correlation was found at initial adaptation (Fig 3B, left), whereas a weak positive correlation was found at initial washout (*r* = 0.36, *p* < 0.01, Fig 3B, right). In anterior force, a weak positive correlation was found at initial adaptation (*r* = 0.43, *p* < 0.01, Fig 3C, left), but none at initial washout (Fig 3C, right).

### Relationship between amount of adaptation and subsequent aftereffect

**Fig 4:**
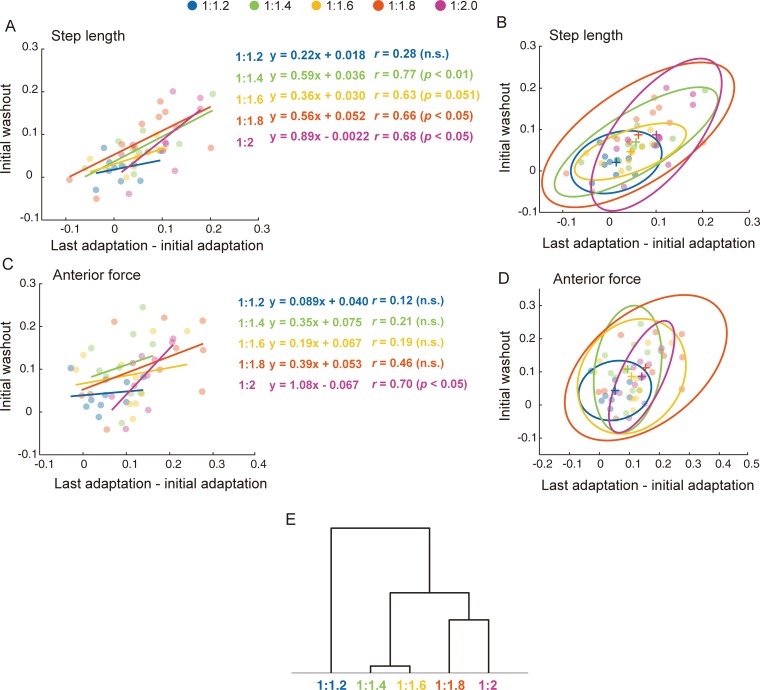
Relationship of the symmetry indices between the extent of adaptation and the initial aftereffect of predictive adapting parameters. **(**A, C): Relationship of the symmetry indices between the extent of adaptation (x-axis) and the initial aftereffect (y-axis) for step length and anterior force, respectively. Each plot indicates individual data in each speed ratio. Regression lines and the correlation coefficients and their significance are denoted. (B, D): The open ellipses represent the 95% confidence ellipse for step length and anterior force in each five speed ratio conditions. Each plot indicates individual data. “+” markers indicates the centers of the ellipses. (E): Dendrogram of the hierarchical clustering analysis (Ward’s method, Euclidean distance) for the data sets of adaptive indices among the five speed ratios.

Fig 4A and 4C shows the relationship between the extent of adaptation and the subsequent aftereffects of the two predictive gait parameters (i.e., step length (Fig 4A) and anterior force (Fig 4C)) in each speed ratio. In step length, a positive correlation between extent of adaptation and aftereffects was found in the 1:1.4, 1:1.8, and 1:2 conditions (1:1.4: *r* = 0.77, *p* < 0.01, 1:1.8: *r* = 0.66, *p* < 0.05, 1:1.4: *r* = 0.68, *p* < 0.05). There are no significant correlations in the 1:1.2 and 1:1.6 conditions (1:1.2: *r* = 0.28, n.s., 1:1.6: *r* = 0.63, n.s.). Regarding anterior force, a positive correlation between adaptation and aftereffects was found only in the 1:2 condition (*r* = 0.77, *p* < 0.01). On the contrary, no significant correlation was found in the other four speed ratios (1:1.2: *r* = 0.12, 1:1.4: *r* = 0.21, 1:1.6: *r* = 0.19, 1:1.8: *r* = 0.46; n.s. in all conditions).

Fig 4B and 4D illustrates the 95% confidence ellipse from the same individual data set of Fig 4A and 4C, respectively. Table 1 shows the characteristic parameters of the ellipses. Almost all the parameters in the 1:1:2 condition were the largest or smallest values (except for the area of the ellipses in step length) in the five speed ratios. Meanwhile, the magnitude relation of the ellipse parameters among the other four speed ratios changed depending on the parameter. Together, the ellipses in 1:1.2 has three characteristics in comparison with the other ratios: (1) the centers were located in the left lower part on the two-dimensional plane, (2) the size was small, and (3) the shapes were nearly precise circles (except comparison with anterior force in 1:1.6). To compare the characteristics regarding the relationship between the amount of adaptation and aftereffect, the five speed ratios were hierarchically clustered based on 14 parameters of the regression lines and ellipses (Fig 4E). The 1:1.2 condition and the other ratios were divided into two principally different clusters.

**Table 1:** Characteristic parameters of the 95% confidence ellipses represented in Fig 4E.

	Step length					Anterior force				
Speed ratio	Major axis length	Center of ellipse		Ellipticity	Area of ellipse	Major axis length	Center of ellipse		Ellipticity	Area of ellipse
		Learning	Aftereffect				Learning	Aftereffect		
1:1.2	0.21	0.015	0.021	0.68	0.022	0.21	0.052	0.044	0.73	0.03
1:1.4	0.41	0.057	0.070	0.34	0.046	0.39	0.094	0.11	0.57	0.07
1:1.6	0.26	0.045	0.047	0.39	0.020	0.39	0.11	0.086	0.82	0.10
1:1.8	0.49	0.062	0.087	0.45	0.085	0.61	0.16	0.11	0.57	0.16
1:2	0.43	0.10	0.088	0.42	0.060	0.38	0.14	0.085	0.37	0.04

## Discussion

The purpose of this study was to characterize gait adaptation process in accordance with the split-belt speed ratio between the left and right sides. We mainly focused on the relative contribution of the two forms of adaptation strategies (reactive feedback control and predictive feedforward control) on split-belt treadmill adaptation. We here tested the learning processes during split-belt walking adaptation under five different speed ratios of the split-belt. The present results showed that a reactive controlled gait parameter (stance time) exhibited quick adjustments just after imposing the split-belt walking and the adjusted pattern was sustained throughout the split-belt condition and then quickly returned to the baseline when returning to tied-belts in the washout period (Fig 2A). The extent of the reactive adjustment had strong linear correlation with the speed ratio of the split-belt (Fig 3A). Meanwhile, the predictive controlled gait parameters (step length and anterior force) showed a typical pattern of adaptation and subsequent aftereffects except for the 1:1.2 condition (Fig 2B and 2C).

Our results clearly showed that the predictive feedforward and reactive feedback control strategies have different responses to changes in the speed ratio of the split-belts. Reactive gait parameter (stance time) showed quick adjustment at the initial split-belt walking period in all five speed ratio conditions, and the adjusted pattern was sustained throughout the adaptation condition and then quickly returned to their baseline level in the tied-belt condition (Fig 2A). Additionally, the extent of the initial adjustment in response to split-belt walking shows significantly strong correlation with the speed ratio of the split-belt (Fig 3A). Even in the smallest speed difference condition (1:1.2), reactive responses were clearly observed and scaled according to the perturbation size. These results are in agreement with the previous results of the split-belt walking study in spinalized cats and intact cats [21], which showed that phase duration (swing time and stance time) alters in response to the speed differences of the split-belt with strong linear correlation. Additionally, bilateral phase adjustments are preserved following spinal cord transection [21], suggesting that such adjustments are mediated by the neural circuits within the spinal cord, at least in cats. In humans, with regard to the neural mechanisms underlying reactive feedback adjustment, neural circuits at the lower level of the CNS, such as the spinal cord and the brain stem, seem to be largely involved [6].

Assuming that the lower CNS is involved in reactive feedback adjustment, the reactive response is most likely controlled by sensory inputs from the periphery that project to spinal locomotor central pattern generators (CPGs), which generate the timing and pattern of locomotor muscle activities [32–35]. A previous study modeled the adjustment of locomotion phases and cycle durations based on data obtained from fictive locomotion in decerebrate cats [36]. Their CPG model consisted of two mutually inhibitory half-centers: one for flexion and one for extension. In the CPG model, locomotor phase durations and step cycle periods can be regulated due to the extensor half-center that is unevenly receiving more sensory inputs regarding foot contact and weight support. Thus, locomotor phases can be adjusted to perturbations through sensory inputs without any cortical control. Regarding specific sensory signals that trigger reactive feedback adjustment, loading input could be involved. A study in human infants stepping showed that load-related feedback could increase the proportion of the extension duration to the cycle duration [37]. In human split-belt walking, the vertical force was higher in the slow leg than that in the fast leg and stance time was longer in the slow leg than that in the fast leg in parallel with vertical force [7]. As these parameters were almost constant throughout the split-belt walking task [7], these are considered to be reactive adjusted parameters. Therefore, we speculate that load-related inputs have an important role for the induction of reactive feedback adjustment especially in the stance time change observed in the present study.

In contrast with the reactive control, the predictive controlled gait parameters (step length and anterior force) showed a clear pattern of adaptation and subsequent aftereffects except for the 1:1.2 condition (Fig 2B and 2C). There were no strong linear relationships between the speed ratio of the split-belt and the extent of responses at initial adaptation and initial washout (Fig 3B and 3C). These results strongly suggest that the control mechanisms of predictive feedforward adaptation are distinct from those of reactive feedback adjustment. In a modeling study on arm reaching, a model predicted that reaching adaptation to visual perturbations should be a nonlinear function of error size [38]. This non-linear adaptation model also explained the published data on saccadic gain adaptation, reaching adaptation to visuomotor rotations, and force perturbations [38]. Although the neural mechanisms of the upper and lower limbs are not considered to be the same, the use of similar nonlinear strategies in locomotor adaptation would explain the present result that the extent of aftereffects was not strongly correlated to the perturbation size.

Concerning the neural mechanisms underlying predictive feedforward adaptation, many previous studies suggested the importance of the cerebellum. Morton and Bastian [9] previously demonstrated that reactive control was not affected in individuals with cerebellar damage, but predictive feedforward adaptation was affected. Similarly, human transcranial magnetic stimulation (TMS) [39] and transcranial direct current stimulation (tDCS) [40] studies showed that cerebellar function are largely involved in the predictive control of locomotor adaptation.

Generally, it is well known that the cerebellum plays an important role in motor learning by processing sensory information and modifying ongoing movement patterns [28,29]. Ataxic patients with severe cerebellar damage demonstrate deficits in proprioception [41,42]. Such impaired perception can lead to poorer detection of errors, which is used in error-based feedforward adaptation [43]. Hoogkamer et al. [12] recently examined sensory perception during split-belt walking and demonstrated that the detection of speed differences of the split-belt was insufficient in severely affected cerebellar patients. Their study also showed that the perception threshold in healthy participants is about 1:1.2. Fig 2B and 2C shows the typical pattern of adaptation and subsequent aftereffects except for the 1:1.2 condition. In addition, cluster analysis based on the relationship between the size of adaptation and the aftereffect showed that the feedforward predictive adaptation process under the ratio of 1:1.2 can be distinguished from those under the other ratios (Table 1, Fig 4). Given the fact that predictive feedforward adaptation occurred under conditions exceeding the average of perception threshold for healthy adults, our results suggest that error detection through sensory perception may drive predictive motor adaptation. Regarding the relationship between sensory perception and motor adaptation during split-belt walking, Vazquez et al. [44] demonstrated that split-belt treadmill adaptation leads to changes in the specific type of perturbations (i.e., left-right difference in belt speed but not position or force). They reported that the perceptual change in leg speed concurrent with motor adaptation was specific to the trained direction (i.e., backward or forward), and this can account for about half of the motor aftereffect. Based on these results, the authors state that the motor and sensory aspects of locomotor adaptation may be tightly linked and may involve overlapping (error-driven, cerebellum-dependent) mechanisms driving adaptation in the motor and sensory domains. Taken together with the strong linkage between motor and sensory domains in gait adaptation, it is plausible that whether or not participants can sense differences between belt speeds during split-belt treadmill walking critically affects predictive feedforward adaptation. Therefore, our results strongly suggest that perceptually detected error signals may trigger feedforward control for gait adaptation.

Regarding the methodological limitations of this study’s experimental protocol, the present adaptation period was shorter (5 min) than that used in many previous studies (typically, 10–15 min) [1,4,8,18,45]. In our previous study [15], however, the typical adaptation curve and subsequent aftereffect have been confirmed in a short adaptation period (6 min). Similarly, the short adaptation period in this study (5 min) led to obviously predictive motor adaptation in the speed ratio typically used in previous studies (i.e., 1:2). Thus, we are certain that differences in the adaptation processes among the five speed ratio conditions were fairly evaluated in such a short adaption period even if the novel locomotor behaviors were not fully adapted.

Our findings indicate that reactive feedback control occurs at any speed ratio and the extent of the reaction proportionally changes with the degree of the speed ratio of the split-belt. On the contrary, predictive feedforward control is necessary when the difference between the speed of the left and right belts was clearly detected. Regarding these two adaptation processes, the present results, in addition to the time course differences presented in previous studies, provide new knowledge about the relative contribution between the two adaptation strategies in response characteristics to perturbation size (i.e., speed ratio of the split-belt). The findings strongly support the hypothesis that the two adaptation strategies have distinct control mechanisms [1,9]. These conclusions might provide important implications for the construction of rehabilitation regimens for the improvement of asymmetric walking in stroke patients.
